
JOURNAL INFORMATION
==============================
NLM Title Abbreviation: ScientificWorldJournal
Journal ID: ScientificWorldJournal
NLM Journal ID: 101131163
Journal ID: TSWJ

The Scientific World Journal

ISSN: 2356-6140
EISSN: 1537-744X
Publisher: Wiley

ARTICLE INFORMATION
==============================
PMCID: PMC5763709
PMID: 20694448
DOI: 10.1100/tsw.2010.147
Article ID: 485278
Article version: 1
Subjects: Clinical Image

Ashy Ears

Rallis Efstathios *
Kintzoglou Stephanos
Department of Dermatology, Veterans Administration Hospital (NIMTS), Athens, Greece
*Efstathios Rallis: efrall@otenet.grAcademic Editor: Daniel Batlle

Publication date: 2010
Electronic publication date: 2010 Aug 3
Volume: 10
First page: 1530
Last page: 1531
Received 2010 Jun 8; Revised 2010 Jun 21; Accepted 2010 Jun 21
Copyright: Copyright © 2010 Efstathios Rallis and Stephanos Kintzoglou.
Copyright year: 2010
License: This is an open access article distributed under the Creative Commons Attribution License, which permits unrestricted use, distribution, and reproduction in any medium, provided the original work is properly cited.
License URL: https://creativecommons.org/licenses/by/3.0/

Keywords: ashy, ear, alkaptonuria, skin

==============================
Clinical Image
*TheScientificWorldJOURNAL* (2010) 10, 1530–1531
ISSN 1537-744X; DOI 10.1100/tsw.2010.147

TheScientificWorldJOURNAL
www.thescientificworld.com

### Ashy Ears

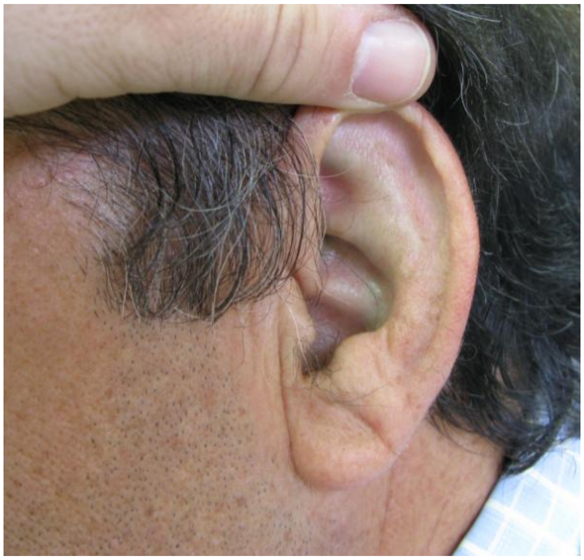

**FIGURE 1**. The (L) ear with blue-gray discoloration.

Efstathios Rallis* and Stephanos Kintzoglou

*Department of Dermatology, Veterans Administration Hospital (NIMTS), Athens, Greece*

**E-mail**: efrall@otenet.gr

*Received June 8, 2010; Revised June 216, 2010; Accepted June 21, 2010; Published August 3, 2010*

**KEYWORDS**: ashy, ear, alkaptonuria, skin

A 56-year-old man was referred to our clinic because of the symmetric appearance of blue-gray discoloration on the helix cartilage of his ears (Figs. 1 and 2). The lesions presented a gradual deterioration during the last 10 years. The rest of his physical examination was unremarkable. According to his past medical history, the diagnosis of alkaptonuria was made at the age of 35 because of the appearance of low back pain and dark urine of approximately 3-years duration. At present, he has experienced arthritis of the spine.

Alkaptonuria is a rare, autosomal-recessive disease of tyrosine degradation resulting from accumulation of homogentisic acid (HGA) within the body due to deficiency of the hepatic enzyme homogentisate 1,2-dioxygenase[1]. *HGD* is the gene encoding homogentisate 1,2-dioxygenase and is the only gene known to be associated with alkaptonuria.

*Corresponding author.
©2010 with author.
Published by TheScientificWorld; www.thescientificworld.com

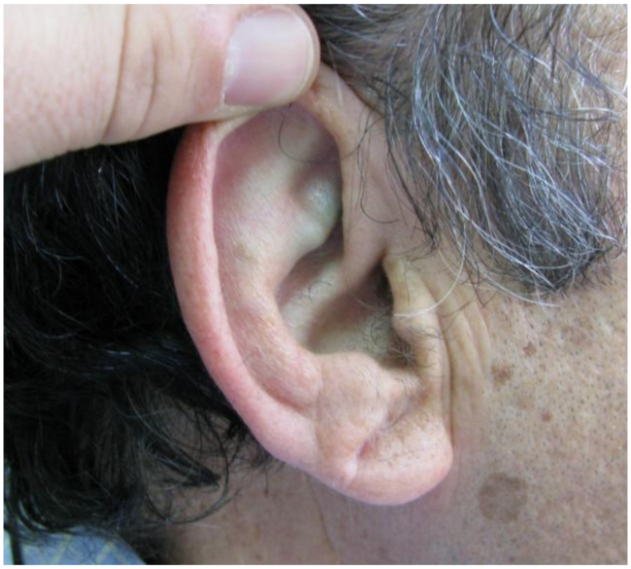

**FIGURE 2**. The (R) ear with blue-gray discoloration.

HGA is oxidized to a pigment-like polymeric material responsible for the black color of urine that is deposited in the cartilage of the body, including ears[2]. Joint symptoms involving the spine are usually seen in the 3rd and 4th decade, while pigment depositions begin to appear approximately in the 4th decade of life[2,3].

A variety of therapeutic modalities have been investigated; however, no preventive or curative treatment has been yet approved for alkaptonuria[3]. Young individuals should be counseled for physical therapy to promote optimal muscle strength and flexibility, but they should avoid contact sports. Dietary decrease of phenylalanine and tyrosine intake has been proposed to reduce the production of HGA. High-dose vitamin C is usually recommended to decrease the deposition in cartilaginous tissues, but has no effect on HGA excretion[4]. Nitisinone has been proposed for the pharmacologic treatment of alkaptonuria[5]. It is a triketone herbicide that inhibits the enzyme that produces HGA and has been approved for the treatment of tyrosinemia type I. Further investigations to determine the potential benefit of nitisinone in alkaptonuria for slowing the progression of joint disease are currently in progress[3].

### REFERENCES

1. Turgay, E., Canat, D., Gurel, M.S., et al. (2009) Endogenous ochronosis. *Clin. Exp. Dermatol*. **34,** e865–868.
2. Roth, K. (2010) Alkaptonuria. Available at: http://emedicine.medscape.com/article/941530. Last accessed: June 3, 2010.
3. Introne, W.J., Kayser, M.A., Gahl, W.A. (2003) Alkaptonuria. In *GeneReviews* [Internet]. Pagon, R.A., Bird, T.C., Dolan, C.R., and Stephens, K., Eds. University of Washington, Seattle. 1993–2003 May 9 [updated 2009 Jul 2]
4. Wolff, J.A., Barshop, B., Nyhan, W.L., et al. (1989) Effects of ascorbic acid in alkaptonuria: alterations in benzoquinone acetic acid and an ontogenic effect in infancy. *Pediatr. Res*. **26,** 140–144.
5. Anikster, Y., Nyhan, W.L., and Gahl, W.A. (1998) NTBC and alkaptonuria. *Am. J. Hum. Genet.* **63,** 920–921.

**This article should be cited as follows:**

Rallis, E. and Kintzoglou, S. (2010) Ashy ears. *TheScientificWorldJOURNAL* **10**, 1530–1531. DOI 10.1100/tsw.2010.147.
